# Umbilical Hernia

**Published:** 1865-12

**Authors:** 


### UMBILICAL HERNIA; SLOUGHING OF FOUR INCHES OF THE SMALL INTESTINES; COMPLETE RECOVERY.

The following case, which occurred in the practice of Dr. Nolan of Wicklow, was read by Dr. Benson, at a meeting of the Surgical Society of Ireland. James Delany, a man about 50, was admitted into Wicklow Infirmary on June 16th. He had an umbilical hernia of about a twelvemonth's standing. Eight days before admission, in struggling to hold a pig, he felt something give way at the tumor, was seized with weakness, followed by pain, and soon after had vomiting. In this state he continued for seven days, using such means as his friends suggested, till the seventh day, when the medical officer of the district was called to visit him. Seeing the state of the case, and the man being in a remote part of the district, he recommended him to be conveyed to the infirmary, to which he was brought on a car, a distance of about seven miles. Dr. Nolan saw him on the eighth day, and found a hernia at the umbilicus about the size of a largish orange, a black mass, with a line of separation forming at the base, and a blush of redness in the surrounding integuments, especially towards the left side; the pulse was weak, the countenance pale and anxious, the stomach gulping up every thing. Manual interference was out of the question. He therefore determined to leave to the adhesive process the repair of the local damage, and to allay irritation and support the patient's strength, ordering a grain of opium every fourth hour till the vomiting ceased; beef-tea, and brandy and water in small quantities, and a linseed meal poultice over the tumor. Next day he had slept well, and was free from pain; the vomiting had ceased after the second pill. The opium was discontinued. The beef-tea, etc., had remained on the stomach; the countenance and pulse were improved; the whole of the integuments had sloughed away, disclosing between three and four inches of small intestine completely disorganized and ready to slough, which it did in two days after, followed by a discharge of bilious curdy fluid. The treatment from this time consisted of giving as much beef-tea and brandy and water as he could take, and throwing up an enema daily of strained gruel and milk, which was generally retained till next day. In about a week the opening began gradually to contract; in a fortnight it had closed; the man daily improved in health and strength, the bowels acted naturally when the enemata discontinued, and he was enabled to leave the hospital on July 22d, a month and five days from his admission.—*Dublin Medical Press.*

